# Family planning use and correlates among female sex workers in a community empowerment HIV prevention intervention in Iringa, Tanzania: a case for tailored programming

**DOI:** 10.1186/s12889-021-11426-z

**Published:** 2021-07-12

**Authors:** S. Wilson Beckham, Melissa Stockton, Noya Galai, Wendy Davis, Jessie Mwambo, Samuel Likindikoki, Deanna Kerrigan

**Affiliations:** 1 Johns Hopkins School of Public Health; Department of Health, Behavior and Society, 624 N Broadway HH 757, Baltimore, MD 21205 USA; 2 Gillings School of Global Public Health, Department of Epidemiology, 135 Dauer Dr., University of North Carolina—Chapel Hill, Chapel Hill, NC 27599 USA; 3 Department of Epidemiology, Johns Hopkins School of Public Health, 615 N. Wolfe St, Baltimore, MD 21205 USA; 4 Department of Psychiatry, Muhimbili University of Health and Allied Sciences, PO Box 65001, Dar es Salaam, Tanzania

**Keywords:** Sub-Saharan Africa, Female sex workers, Unmet need, Intervention, Contraception

## Abstract

**Background:**

Female sex workers in sub-Saharan Africa face high unmet need for family planning and higher risk for unintended pregnancy. Community empowerment HIV prevention approaches have the potential to increase family planning uptake and present an opportunity to integrate HIV, reproductive health, and contraception. This article describes family planning use and pregnancy among female sex workers in Iringa, Tanzania and evaluates whether engagement in a community empowerment HIV prevention program is associated with contraceptive use.

**Methods:**

This study consists of secondary analysis from a two-community randomized controlled trial following a longitudinal cohort over 18 months. We implemented a year-long community empowerment intervention consisting of 1) a community-led drop-in-center; 2) venue-based peer education, condom distribution, and HIV testing; 3) peer service navigation; 4) sensitivity trainings for providers and police; and 5) text messages to promote engagement. Additionally, monthly seminars were held at the drop-in-center, one of which focused on family planning. Modified Poisson regression models were used to estimate the association between program exposure and family planning use in the intervention arm. (Trials Registration NCT02281578, Nov 2, 2014.)

**Results:**

Among the 339 participants with follow-up data on family planning, 60% reported current family planning use; 6% reported dual use of modern contraception and condoms; over 90% had living children; and 85% sought antenatal care at their most recent pregnancy. Among the 185 participants in the intervention arm, the adjusted relative risk (aRR) of family planning use among female sex workers who reported ever attending the Shikamana drop-in-center and among female sex workers who reported attending a family planning-related workshop was respectively 26% (aRR 1.26 [95% Confidence Interval (CI): 1.02–1.56]) and 36% (aRR 1.36 [95%CI: 1.13–1.64) higher than among those who had not attended.

**Conclusion:**

There is a clear need for family planning among this population. General program exposure and exposure to a family planning workshop were associated with higher family planning use, which suggests that community empowerment models have potential to increase family planning uptake for this vulnerable group.

## Introduction

Female sex workers (FSW) in sub-Saharan Africa (SSA) are at heightened risk for unintended pregnancy as well as for HIV and other sexually transmitted infections (STI), demonstrating a high unmet need for family planning (FP) [1, 2]. A recent global literature review indicated that FSW experience high rates of unmet need for family planning and safe conception services, unintended pregnancies, and abortion, and that they practice inconsistent condom use due to restrictive policy environments, stigma and discrimination in health care settings, gender inequality, and economic marginalization [3]. While evidence from SSA is limited, studies in Zambia, Uganda, and Cote D’Ivoire have shown that unintended pregnancy among FSW is common [4–6]; in Zambia, more than half of surveyed FSW reported at least one unplanned pregnancy [4]. Qualitative research in Ethiopia found that missed injections, skipped pills, and inconsistent condom use were causes of unintended pregnancy among FSW [7]. A recent study in Tanzania among FSW living with HIV found that while most wanted to prevent pregnancy, only 4% were using dual methods (condoms plus modern contraception), and only 5% reported using condoms consistently [8]. Program surveillance data in Tanzania found 5.7% dual protection use and 16% consistent condom use among FSW attending services [9]. Once pregnant, these same challenges may constitute barriers to accessing antenatal care (ANC) or prevention of mother-to-child transmission (PMTCT) services [10, 11]. More evidence on the predictors and correlates of family planning use (or nonuse) of reproductive health (RH) and FP services is needed to ensure the provision of appropriate services that address the burden of unplanned pregnancy among this vulnerable group.

Research and programing for FSW has largely focused on HIV and STI prevention without taking into account their RH and FP needs [3, 12]. FSW’s family planning needs are similar to other women of reproductive age, but they also present unique challenges due to heightened HIV/STI risks, multiple partnership types with whom they may have differing fertility desires, inability to control condom use in some situations, and constrained access to healthcare or FP services [3, 10, 13–15]. The evidence base of HIV prevention interventions for FSW is substantial [16], but RH and FP services tailored for the needs of FSW are urgently needed, particularly in low- and middle- income countries [17].

Incorporating RH and FP services into existing HIV services or leveraging HIV prevention efforts to provide FP for FSW could potentially improve FP use and reduce unintended pregnancies [5, 18]. While HIV prevention interventions are often not specifically designed to improve family planning use, FP is increasingly incorporated into HIV care for those living with HIV [19], as HIV testing and PMTCT services are similarly already integrated into antenatal care (ANC) in SSA [20]. Furthermore, the World Health Organizations’ (WHO) four-prongs of PMTCT (HIV prevention; prevention of unintended pregnancies; HIV treatment during pregnancy, labor, and breastfeeding; and caring for women and their children and families) inherently intertwines HIV and reproductive health services [21]. Embedding tailored reproductive health services that are sensitive to FSW’s RH needs within HIV programming may be an effective means of providing RH services.

Community empowerment models for HIV prevention are effective at reducing the odds of HIV infection and improving condom use among FSW [22]. Such models often take a multi-pronged approach to HIV prevention, including elements of community-led mobilization to address social and structural barriers (such as stigma) to HIV prevention, treatment and care, as well as to provide peer education and service navigation, condom distribution, and HIV/STI screening. As sexual and RH knowledge and access to care and contraception all influence FP uptake, community empowerment approaches could theoretically improve FP use. In Tanzania, a two-community randomized control trial (RCT) was conducted to evaluate a community empowerment HIV prevention program [23, 24]. Using data from this trial, this article seeks to describe family planning use and pregnancy among a population of FSW in Iringa, Tanzania, investigate any differences by HIV status, and evaluate whether engagement in the Shikamana intervention was associated with modern contraceptive use.

## Materials and methods

This manuscript describes secondary analysis of data from the 18-month follow-up of the Project Shikamana cohort of FSW in Iringa, Tanzania. The project involved a community-randomized trial of a community empowerment-based HIV prevention intervention in two communities in the Iringa region of Tanzania (NCT02281578, Nov 2, 2014). Randomization occurred at the community level, with one community randomized to receive the intervention. The intervention components included 1) a community-led drop-in center (DIC) creating a safe space that facilitated social cohesion and mobilization activities; 2) venue-based peer education, condom distribution, and HIV testing; 3) peer service navigation and social support for HIV-infected participants; 4) sensitivity trainings for HIV providers and police; and 5) text messages to promote engagement in the intervention, and adherence to clinic appointments and antiretroviral therapy (ART) adherence for FSW living with HIV [24].

The baseline bio-behavioral survey was conducted from October 2015 to April 2016. The intervention was then implemented in one community for 18-months, at which point the post-intervention survey was conducted. Both surveys gathered information on demographics, reproductive histories and family planning use, sexual risk behaviors (e.g., condom use), work-related risks (e.g., number of clients), and HIV-related information (serostatus, knowledge, history of testing, and health service use for HIV-infected participants). The follow-up survey captured program exposure and engagement in both communities, not limited to the intervention community.

At the DIC, monthly seminars were held, with topics chosen by members of the intervention community. Seminar themes included gender-based violence, family planning, sex work stigma and human rights, HIV/STI prevention, and income generation. The FP session was run by an outreach nurse, who was also available to provide FP methods at the session (e.g., injectable contraception). Following some seminars, participants decided to organize regular weekly meetings at the DIC on specific topics, including family planning, to continue to learn from each other, discuss problems, and identify solutions as a sex worker community. The DIC was also open during the week for walk-ins and staffed by counselors who offered HIV counseling and testing and peer educators who gave education and distributed condoms. Further details on the intervention and characteristics of the baseline cohort as well as the main outcomes of the trial have been previously published [23, 24].

### Sample

Time-location sampling at entertainment venues (bars, hotels, etc.) was employed to achieve a sample of at least 200 HIV-infected FSW. The total baseline sample included 496 participants, with 254 in the intervention community. Individuals who did not complete the 18-month follow-up survey (*n* = 109), who did not have complete FP data at baseline and follow-up (*n* = 3), and who were pregnant at follow-up (*n* = 37) were excluded from this analysis. An additional *n* = 8 participants had conflicting answers on FP use (*n* = 7 reported female sterilization at baseline and a long-acting reversible FP method at follow-up, *n* = 1 reported both injectables and pills at follow-up), and so were dropped from the analysis, leaving a total of 339 participants. Two of the 339 participants were missing data on program exposure, so were dropped from the multivariable analysis but included in other analyses. Further information on the recruitment and sampling methods are described elsewhere [23].

### Variables

#### Family planning use

The primary outcome was self-reported current family planning use measured at the 18-month follow-up survey. Participants were asked if they currently used various modern contraceptive methods including injectable, implant, oral pill (combined or progesterone only), tubal ligation, or male or female condoms as birth control. These represent a complete list of modern FP methods available in Tanzania at the time. “Current family planning use” was defined as use of at least one of the aforementioned modern methods.

#### Program exposure

Program exposure was measured at the 18-month follow-up survey. General program exposure was dichotomized any/none and was defined as at least one of the following: 1) any attendance of the monthly seminars, participant-organized weekly meetings, or walk-in visits held at the Shikamana DIC; 2) obtaining condoms at the DIC, or 3) getting tested for HIV at the DIC. Family planning program exposure was defined as attendance of a seminar, workshop, or meeting specifically about FP.

#### Reproductive and family planning history

Additionally, the baseline survey captured historical FP use (e.g., whether participants had ever used any of the modern contraceptives). The 18-month follow-up also asked participants about their reproductive histories (numbers of lifetime pregnancies and living children; current pregnancy status; current pregnancy intentions, intendedness of most recent pregnancy; and age at first pregnancy) and use of ANC services (sought ANC for recent pregnancy; location of care). Since formative qualitative work previously indicated that some FSW were not accessing ANC services because they were not accompanied by husbands for couples HIV testing [10], the survey also asked if participants were accompanied by a male partner, told to bring a partner, or did not or could not access services due to inability to bring a partner.

### Analysis

Frequencies and proportions are reported for basic demographics and FP use (Tables 1 & 3). Reproductive histories by HIV status (Table 2) and demographics by current FP use (Table 4) were calculated using Pearson’s Chi-square. For Table 5, to simultaneously evaluate the effect of various factors on the outcome, we have applied modified Poisson regression models with robust standard errors. This method had been proposed as an alternative to logistic regression for binary data in cases where the proportion of the outcome is high, as is the case for family planning in our cohort [25]. Two separate modified Poisson regression models with adjusting for clustering by venue were used to estimate the association between the outcome variable, current FP use, and 1) any program exposure, and 2) exposure to the FP workshop, controlling for potential confounders that were theorized to be related to the outcome and/or exposure. Crude models were run, then models using stepwise backwards elimination adjusted for all potential confounders or all factors that were associated (*p*-value< 0.1) with the current FP use in bivariate analysis. The final models retained variables significant at *p*-value< 0.05. We used Stata 16 SE (StataCorp, College Station, Texas) for analyses.

**Table 1:** Sample Demographics (*n* = 339)

	Comparison n (%)
Age	
≤ 30	186 (54.87)
> 30	153 (45.13)
Community	
Intervention	185 (54.57)
Comparison	154 (45.43)
Education	
None/some primary	241 (71.09)
Some secondary +	98 (28.91)
Relationship status	
Single/divorced/widowed	194 (57.23)
Married/partnered	145 (42.77)
Ethnicity ^a^	
Groups not local to the region	139 (41.12)
Local groups (Hehe, Bena)	199 (58.99)
HIV serostatus	
Positive	174 (51.33)
DIC attendance	
Ever Attended Shikamana Center ^b^	92 (27.14)
FP programming exposure	
Attended FP Workshop or Meeting ^b^	57 (16.91)

^a^ Refused to answer *n* = 1; ^b^ Missing *n* = 2

**Table 2:** Reproductive Histories of FSW by HIV status, after 18-months of Follow-up

	Total n (%)	HIV-negative n (%)	HIV-positive n (%)	*P*-value ^a^
**Reproductive histories (*n*** **= 339)**				
Number of lifetime pregnancies				0.122
0	18 (5.31)	10 (6.06)	8 (4.60)	
1 or 2	162 (47.79)	87 (52.73)	75 (43.10)	
> 3	159 (46.90)	68 (41.21)	91 (52.30)	
Currently trying to become pregnant ^b^				0.754
No	265 (78.17)	127 (76.97)	138 (79.31)	
Yes	71 (20.94)	36 (21.82)	35 (20.11)	
**History of pregnancy (*n*** **= 321)**				
Living children ^c^				0.812
0	7 (2.18)	4 (2.58)	3 (1.81)	
1 to 2	225 (70.09)	109 (70.32)	116 (69.88)	
3+	77 (23.99)	35 (22.58)	42 (25.30)	
Most recent pregnancy was planned/intended				0.651
No	174 (54.21)	82 (52.90)	92 (55.42)	
Yes	147 (45.79)	73 (47.10)	74 (44.56)	
Age at first pregnancy ^d^				0.305
< 18	101 (31.46)	43 (27.74)	58 (34.94)	
> 18	212 (66.04)	107 (69.03)	105 (63.25)	
Sought ANC for most recent pregnancy				0.712
No	48 (14.95)	22 (14.19)	26 (15.66)	
Yes	273 (85.05)	133 (85.81)	140 (84.34)	
**ANC attenders for most recent pregnancy (*n*** **= 273)**				
Location of ANC services ^e^				0.735
Public large hospital	182 (66.67)	88 (66.17)	94 (67.14)	
Public medium or small facility	70 (25.64)	34 (25.56)	36 (25.71)	
Private clinic	20 (7.33)	11 (8.27)	9 (6.43)	
Told to Bring Husband/Partner to ANC				0.088
No, my partner was with me	20 (7.33)	10 (7.52)	10 (7.14)	
No, that didn’t happen	20 (7.33)	5 (3.76)	15 (10.71)	
Yes	233 (85.35)	118 (88.72)	115 (82.14)	
No ANC services because no husband/partner (*n* = 232)				0.457
No	194 (83.26)	100 (84.75)	94 (81.74)	
Yes	38 (16.31)	17 (14.41)	21 (18.26)	

^a^ Chi-squared; ^b^ Missing n = 3; ^c^ Missing *n* = 12; ^d^ Missing n = 8; ^e^ Missing *n* = 1

### Ethical statement

Institutional review boards at the Johns Hopkins Bloomberg School of Public Health (USA, FWA#0000287), and National Institute of Medical Research (Tanzania), and Muhimbili University of Health and Allied Sciences (Tanzania) gave ethical approval for this study, which was performed following the principles stated in the Declaration of Helsinki [26]. All participants gave oral informed consent before participation.

## Results

### Participant characteristics

Of the 339 participants who completed both the baseline and the 18-month follow-up survey, less than half the participants were over 30 years old (Table 1). Around 55% of participants were from the intervention community. Educational attainment was low with less than 30% of participants achieving some secondary education. Over half of participants were single, divorced, or widowed. Nearly 60% of participants were members of the local Hehe or Bena ethnic groups. Just over half of participants were living with HIV. Nearly 30% of participants had ever attended the Shikamana center and only around 17% of participants had ever attended a specific family planning session.

### Bivariate analysis of reproductive histories by HIV status

Most women (95%) had experienced at least one pregnancy over their lifetime (Table 2). HIV serostatus was not significantly associated with any of the reproductive history indicators (*p*-values> 0.05). Of those not currently pregnant, about a fifth were currently trying to become pregnant. Among those who had experienced at least one pregnancy over their lifetime, over 90% had living children. However, over half of women reported that their most recent pregnancy was unplanned and a third of women were < 18 years old at the time of their first pregnancy. Around 85% of women sought ANC at their most recent pregnancy and over 90% of those sought ANC from a public facility. The primary reason for not seeking ANC was abortion (*n* = 21) or miscarriage (*n* = 22). Among women who sought ANC, 85% (*n* = 233) were told to bring a husband or partner and of those 233, 16% did not then access ANC because of their lack of husband/partner to accompany them.

### Prevalence of family planning use

Nearly 84% of participants reported ever using modern FP (Table 3). Of all study participants, 61% reported current modern family planning use, though 69% of participants who reported that they were *not* currently trying to become pregnant at the follow-up survey were currently using contraception. As such, over 31% of participants not currently trying to become pregnant demonstrate unmet need for family planning. Participants used an array of FP methods. Male condoms were the most frequently reported type of family planning method ever used, although less than 20% of participants reported current use of male condoms at the follow-up survey. Almost one third of participants reported ever using injectable family planning methods and just over 20% of participants reported current use of injectables. Dual use of contraception, defined as use of both one modern method *and* condom (either male or female), was low, around 6%.

**Table 3:** Modern Family Planning Use

	Total sample (*n* = 339)n (%, [95% CI])	Not trying to get pregnant (*n* = 265) n (%, [95% CI])
**Family Planning Use**		
Ever ^a^	283 (83.48 [79.09–87.27])	–
Current ^b^	208 (61.36 [55.94–66.57])	184 (69.43 [63.50–74.92])
**Method Type**		
Injectables		
Ever	101 (29.79 [24.97–34.97])	–
Current	72 (21.24 [17.01–25.98])	67 (25.28 [20.16–30.96])
Implants		
Ever	53 (15.63 [11.94–19.95])	–
Current	50 (14.75 [11.14–18.98])	46 (17.36 [13.00–22.47]])
Pills (any type)		
Ever	38 (11.21 [8.05–15.06])	–
Current	15 (4.42 [2.50–7.19])	11 (4.15 [2.09–7.31])
Intra-Uterine Device (IUD)		
Ever	13 (3.83 [2.06–6.47])	–
Current	5 (1.47 [0.48–3.41])	5 (1.89 [0.62–4.35])
Female Sterilization		
Ever	4 (1.18 [0.32–3.00)	–
Current	4 (1.12 [0.32–3.00])	4 (1.51 [0.41–3.82])
Male Condoms		
Ever	211 (62.24 [56.84–67.42])	–
Current	65 (19.17 [15.12–23.78])	53 (20.00 [15.36–25.33])
Female Condoms		
Ever	18 (5.33 [3.19–8.29])	–
Current	18 (5.31 [3.18–8.26])	14 (5.28 [2.92–8.71])
Dual (condom + modern method)		
Current	21 (6.19 [3.88–9.31])	16 (6.04 [3.50–9.62])

^a^ Ever use measured at baseline; ^b^ Current use measured at 18 months

### Bivariate analysis of modern family planning use

Community, number of clients a week, and history of FP use were significantly associated with current FP use (Table 4). Of those currently using FP, nearly 60% of participants were from the intervention community, whereas more than half of those not currently using family planning were from the comparison community. About 45% of participants had two or more clients a week. A larger proportion of those currently using family planning (52%) had two or more clients a week compared to those not currently using family planning (35%). Almost all (*n* = 192) of the participants who reported current FP use also reported ever use at baseline. Sixteen of the 56 participants (29%) who stated they had never used family planning at baseline reported current use at the 18-month follow-up survey. Almost half of participants reported inconsistent condom use over the past 30 days.

**Table 4:** Bivariate correlates of current FP use at 18-month Follow-Up (*n* = 339)

	Total n (%)	No current FP use (*n* = 131)	Current FP use (*n* = 208)	*P*-value ^a^
**Community (study arm)**				0.093
Intervention	185 (54.57)	64 (48.85)	121 (58.17)	
Comparison	154 (45.43)	67 (51.15)	87 (41.83)	
**Demographics**				
Age				0.519
≤ 30	186 (54.87)	69 (52.67)	117 (56.25)	
> 30	153 (45.13)	62 (47.33)	91 (43.75)	
Education				0.975
None/some primary	241 (71.09)	93 (70.99)	148 (71.15)	
Some secondary +	98 (28.91)	38 (29.01)	60 (28.85)	
Relationship status				0.657
Single/divorced/widowed	194 (57.23)	73 (55.73)	121 (58.17)	
Married/partnered	145 (42.77)	58 (44.27)	87 (41.83)	
Ethnicity ^b^				0.916
Non-local groups	139 (41.00)	53 (40.46)	86 (41.35)	
Local groups (Hehe, Bena)	199 (58.70)	77 (58.78)	122 (58.65)	
HIV serostatus				0.957
Negative	165 (48.67)	64 (48.85)	101 (48.56)	
Positive	174 (51.33)	67 (51.15)	107 (51.44)	
**Work-Related Risk Factors**				
# Clients per week				**0.002**
0–1	184 (54.28)	85 (64.89)	99 (47.60)	
2+	155 (45.72)	46 (35.11)	109 (52.40)	
Consistent condom use (CCU) (30 days)				0.251
No	166 (48.97)	59 (45.04)	107 (51.44)	
Yes	173 (51.03)	72 (54.96)	101 (48.56)	
Venue type				**0.036**
Local bar & other	220 (64.90)	94 (71.76)	126 (60.58)	
Modern bar	119 (35.10)	37 (28.24)	82 (39.42)	
**Reproductive History Factors**				
Lifetime pregnancies				0.585
0–2	180 (53.10)	72 (54.96)	108 (51.92)	
3+	159 (46.90)	59 (45.04)	100 (48.08)	
Previously used modern FP method (ever at baseline)				**< 0.001**
No	56 (16.52)	40 (30.53)	16 (7.69)	
Yes	283 (83.48)	91 (69.47)	192 (92.31)	

^a^ Chi-squared; ^b^ Refused to answer *n* = 1; Bold = significant at *p* < 0.05 level

**Table 5:** Relative Risk of Current Family Planning Use by Program Exposure (*n* = 185)^a^

	Unadjusted Relative Risk Ratio RR (95% CIs)	*p*-value	Adjusted Relative Risk Ratio aRR (95% CIs) ^b^	*P*-value
Any Program Exposure at Shikamana Center	**1.30 (1.05–1.61)**	**0.018**	**1.26 (1.02–1.56)**	**0.029**
Family Planning Program Exposure	**1.43 (1.18–1.73)**	**< 0.001**	**1.36 (1.13–1.64)**	**0.001**

^a^ Intervention community only; Missing n = 2; Initial stepwise backward modified Poisson logistic regression models with robust standard errors, controlling for number of clients per week and history of modern FP use at baseline; final model included history of modern FP use at baseline

### Multivariable analysis of program exposure and FP use

Of the participants in the intervention community, two had missing data on program exposure. The adjusted relative risk of current FP use among FSW who reported ever attending the Shikamana center compared to those who never attended the Shikamana center was aRR 1.26 (1.02–1.56, *p* = 0.029). The adjusted relative risk of current FP use among FSW who reported attending the FP-related workshop at the Shikamana center compared to those who did not attend the FP-related workshop was aRR 1.36 (1.13–1.64, *p* = 0.001).

## Discussion

This study provides an overview of the reproductive health profile of this population of FSW. Among the study population, reproductive histories and fertility preferences did not significantly vary by HIV status, which indicates unmet service needs for both FSW who are uninfected (prevention of HIV, prevention of unwanted pregnancy) and FSW who are infected (safer conception, prevention of unwanted pregnancy, prevention of transmission to partners). ANC care seeking was high; over 85% of participants sought ANC during their last pregnancy. Around 84% of participants had ever used contraception and over 60% were currently using contraception. Community (study arm), number of clients a week, and history of FP use were significantly associated with current FP use. Further, the study provides insights on the potential impact of involvement in a community empowerment HIV prevention intervention – an intervention with a very limited (one seminar) FP component – on FP use. The odds of current FP use among FSW was higher among those who reported ever attending the Shikamana DIC, compared those who had not, and was higher among those who reported ever attending the FP-related workshop, than those who had not.

ANC service seeking during pregnancy was relatively high among participants, though an alarmingly high proportion (85%) were told to bring a male partner; of those told to bring a partner, 16% reported they did not access antenatal care due to their lack of partner or inability to bring him. A qualitative study conducted in Tanzania found that pregnant FSW, like other women, seek ANC services, but often face stigma and discrimination and/or denial of services [10]. Other qualitative research in Tanzania have also found that health providers may deny services to women and adolescents attending antenatal care without a partner [27, 28]. This phenomenon may be due in part to Tanzanian maternal and child health policies, guidelines, and strategies that encourage male involvement in ANC and HIV testing as part of PMTCT [29]. While male participation in ANC care is valuable, the lack of male accompaniment should not constitute an additional barrier to receiving services. The interpretation and implementation of such polices and guidelines at the facility level should be investigated to ensure that all pregnant people receive appropriate care.

Family planning use was high among study participants; nearly 85% of participants reported ever using a modern FP. Many factors may explain FP use and non-use. In this study, community of residence, average number of clients a week, and history of FP use were significantly associated with current FP use. However, unmet need for FP was also high among the study population – over 30% of those not currently trying to get pregnant did not report using a modern method of contraception. A similar 30% of FSW living with HIV in Njombe and Mbeya regions and who did not want to get pregnant in the next 2 years had an unmet need for contraception, as they were neither consistent condom users nor users of an effective non-barrier method [15]. Over a quarter of FSW from a study conducted in Swaziland, Burkina Faso, and Togo had unmet need for family planning [18]. Among a population of Kenyan sex workers not trying to become pregnant within the next year, around 40% were either not using a contraceptive method or only using condoms to prevent pregnancy [30]. Comprehensive interventions that meet the reproductive health needs of key populations such as FSW are needed to address unmet need for family planning [3, 30].

While the most commonly reported contraceptive ever used was condoms (60%), only around 20% reported current use of condoms as an FP method at the follow-up survey and almost half of participants reported inconsistent condom use over the last 30 days. Furthermore, very few participants reported dual use of both a modern contraception method and condom use. Similarly in Swaziland, 16% of FSW were found to be consistent users of condoms alone; 39% used non-barrier modern methods (without consistent condom use); 8% were dual method users; and 38% were inconsistent condom users or used other methods or none [31]. Condom use was much higher in a recent study conducted in Kenya where a total of 98.8% FSW reported using male condoms in the past month, and 64.6% reported using female-controlled non-barrier modern contraception [32]. The low levels of consistent condom use leave FSW vulnerable to both unplanned pregnancy and STI including HIV. Efforts are needed to increase uptake and use of FP among FSW and improve consistency of condom use.

Little is known about fertility preferences among FSW and other marginalized groups of women [33–35]. In this study, about a fifth of participants both living with and without HIV were currently trying to get pregnant. Another study in Tanzania, conducted in neighboring regions to this one, found that a 21% of FSW living with HIV were trying to get pregnant, and another 20% wanted to in the near future. However, they lacked information on safe conception practice [8]. Similarly, a study of Kenyan FSW living with HIV found that 25.5% of FSW wanted to have children and 10.2% were currently trying to have children [36]. Another study conducted in Swaziland, Burkina Faso, and Togo found that nearly a fifth of FSW reported that they were currently trying to conceive [18]. In the Dominican Republic, factors associated with HIV-infected FSW’s fertility desires included being younger, having experienced pregnancy loss, and having higher HIV-related internalized stigma [33]. Fertility preferences may adversely impact HIV prevention and treatment efforts such as higher risk of unprotected sex [36], and treatment interruptions among those living with HIV [34]. Many factors may drive FSW’s fertility preferences. A qualitative study conducted in Tanzania found that FSW sought to become pregnant to gain respect as mothers, to avoid stigma, and/or to solidify relationships [10]. Recognizing FSW’s fertility preferences, appropriate and accessible FP and ANC services are needed to address structural and social barriers to ANC and PMTCT and ensure safer pregnancies, including for women living with HIV [18].

Exposure to the community empowerment program – both in terms of ever attending the Shikamana center as well as ever attended the FP-specific session – were associated with greater odds of current FP use. This finding suggests that community empowerment models of HIV care provision may also positively impact family planning behaviors, potentially through developing sexual and RH knowledge, improving access to condoms and encouraging their use, and reducing barriers to health care seeking in general. The potential for empowerment to improve family planning outcomes has been documented in two recent literature reviews among the general population of women, though findings have been variable, with some showing an effect and others not [37, 38]. However, among FSW very few HIV prevention interventions have evaluated the intervention’s effect on family planning. Even fewer interventions aimed at improving FP use among FSW have been conducted. However, a study evaluating an integrated set of FP and HIV services for FSW in Dar es Salaam [8] and another study on improving adherence to FP in Kenya among FSW are currently underway [17]. A review on the potential effectiveness and feasibility of integrating FP into HIV services found that integrated programs were often associated with higher modern method of contraceptive prevalence and knowledge, though findings were mixed [19].

### Limitations

There are some limitations inherent in this analysis. The specific FP questions assessed here were only measured at follow-up. As such, we were unable to determine whether FP use increased from baseline to follow-up. Furthermore, we were only able to estimate the cross-sectional association between exposure to community empowerment program and current family planning use at follow-up. Thus, we cannot determine causality of community empowerment on family planning use; it may be that women who were more likely to use family planning were more likely to be involved in the drop-in-center activities. That said, we were able to control for ever use of family planning in the regression models, suggesting that FP use did come before intervention exposure. Further study with clear temporality is warranted to determine the true association. This study was a secondary analysis of a larger study; thus, the study was not powered on the outcome, so interpretations should be cautious. As with all self-report data, responses may be subject to social desirability bias. For example, FP use and program exposure may have been over-reported. The time-location sampling methods may limit the generalizability of these findings to venue-based FSW. Future research designed to assess these outcomes could overcome some of these limitations.

## Conclusions

The working environments of FSW in sub-Saharan Africa can place them at higher risk of unintended pregnancy, as well as STI including HIV. Community empowerment-based interventions have been shown to be effective at improving consistent condom use and reducing HIV infection. This study indicates that other health outcomes, such as modern family planning use, may also be impacted by these interventions, especially when family planning is part of a comprehensive empowerment-based program. More attention is needed on integration of family planning into interventions tailored for sex workers and other marginalized groups, including implementation research to test and develop best practices that serve the needs of the communities.

## Data Availability

The dataset analyzed during the current study are available from the corresponding & senior authors upon reasonable request.

## Abbreviations

ANC: Antenatal care
aRR: Adjusted relative risk
ART: Antiretroviral therapy
BL: Baseline
CI: Confidence internal
CCU: Consistent condom use
DIC: Drop-in-center
FP: Family planning
FSW: Female sex worker(s)
HIV: Human immunodeficiency virus
PMTCT: Prevention of mother-to-child transmission
RCT: Randomized controlled trial
RH: Reproductive health
RR: Relative risk ratio
SSA: Sub-Saharan Africa
STI: Sexually transmitted infection(s)
WHO: World Health Organization
